# “Current incidence of injuries in Iran; findings of STEPS survey 2021”

**DOI:** 10.1016/j.heliyon.2023.e20907

**Published:** 2023-10-14

**Authors:** Elnaz Shahmohamadi, Erfan Ghasemi, Esmaeil Mohammadi, Maryam Nasserinejad, Sina Azadnajafabad, Mohammad-Reza Malekpour, Mohammad-Mahdi Rashidi, Naser Ahmadi, Negar Rezaei, Mohammadreza Naderian, Moein Yoosefi, Yosef Farzi, Nazila Rezaei, Rosa Haghshenas, Elham Abdolhamidi, Ali Golestani, Ameneh Kazemi, Mahdi Delaram Dizaj, Niusha Nazari, Azadeh Momen Nia Rankohi, Mahbobeh Darman, Shirin Djalalinia, Alireza Moghisi, Farshad Farzadfar

**Affiliations:** a Non-communicable Disease Research Center, Endocrinology and Metabolism Population Sciences Research Institute, Tehran University of Medical Sciences, Tehran, Iran; b Department of Neurological Surgery, University of Oklahoma Health Sciences Center, Oklahoma, Oklahoma, USA; c Center for Life Course Health Research, Faculty of Medicine, University of Oulu, Oulu, Finland; d Tehran Heart Center, Cardiovascular Diseases Research Institute, Tehran University of Medical Sciences, Tehran, Iran; e Department of Mathematics and Statistics, Memorial University of Newfoundland, St. John's, NL, Canada; f Endocrinology and Metabolism Research Center, Endocrinology and Metabolism Clinical Sciences Institute, Tehran University of Medical Sciences, Tehran, Iran; g Deputy of Health, Ministry of Health and Medical Education, Tehran, Iran; h Development of Research and Technology center, Deputy of Research and Technology Ministry of Health and Medical Education, Tehran, Iran; i Deputy General Director for NCD Management Office, Ministry of Health and Medical Education, Tehran, IR, Iran

**Keywords:** Injuries, Iran, Epidemiology, Road traffic injury, Fall

## Abstract

**Background:**

The updated epidemiology of injuries at the national and sub-national levels are required for policymakers to effectively handle the burden of injuries. This paper aimed to assess the incidence and risk factors of different injuries in Iran based on a recent national survey.

**Methods:**

We used data from Iran Stepwise approach to surveillance (STEPS) Survey 2021, a population-based study in urban and rural areas of Iran's 31 provinces. A multistage clustered probability design and weighting adjustments were used to select eligible individuals and generate estimations. We estimated the incidence of injuries, assessed sociodemographic variables, and identified potential behavioral risk factors associated with injuries, and results were reported for sociodemographic and geographic stratifications.

**Result:**

Data from 27,874 participants of the STEPS survey were assessed, of which 1538 (5.5 %, 95 % CI: [5.2–5.8]) reported having an injury in the past 12 months. Falls (44.4 %) were the most common cause of injury, followed by road traffic injury (21.7 %) and exposure to mechanical forces (16.5 %). Except for falls and burns, males had a higher proportion of all types of injuries. Logistic regression analysis showed that being male (OR: 1.7, [1.5, 2.0]) and being an occasional or heavy alcohol drinker (OR: 2.0, [1.3, 3.0] and OR: 2.7, [1.7, 4.1] respectively) were significant risk factors associated with road traffic injuries. Seatbelt use was 90.0 % among both drivers and front-seat passengers, while the use of safety car seats for children was as low as 9.4 %. Injury incidence varied significantly among provinces, with the highest incidence among males observed in Razavi Khorasan (11.2 %) and among females observed in Tehran (12.0 %).

**Conclusion:**

This study investigated the updated epidemiology of injuries in Iran and revealed socioeconomic and geographic disparities across country. This epidemiological information can be used to modify injury prevention programs.

## 1 Introduction

Every year, injuries claim the lives of millions of people since at least 7 % of the world's deaths are caused by injuries, making them a health threat in every country and a significant issue on a global scale [1]. The burden of nonfatal health outcomes is considerably more remarkable, as a significantly larger frequency of injuries can lead to long-term disability and financial loss [2]. The global injury burden is unequally distributed, with the majority of injuries occurring in low- and middle-income countries due to a lack of safety measures and healthcare infrastructure [3]. According to the Global Burden of Diseases (GBD) and Injuries study in 2019, injuries are one of the leading causes of combined death and morbidity in Iran [1].

Road traffic injuries (RTIs), falls, burns, drowning, poisonings, self-harm, and interpersonal violence are the leading causes of injury that result in death or disability worldwide [4]. When comparing different types and external causes of injuries in the field of epidemiology, factors such as sex, age, income, and geography should be considered as contributors to variations in burden of disease/injury. Moreover, it is essential to identify and target high-risk populations, such as individuals who engage in risky behaviors [5]. Even though Iran has a mandatory seatbelt and helmet legislation and alcohol consumption is prohibited by law, risky behaviors including drunk-driving, speeding, and refusing to wear helmets, seatbelts, and car seats for children continue to be risk factors for different injuries among Iranian people [[5], [6], [7]].

Although previous research has estimated the burden of various communicable and non-communicable diseases (NCDs), data on different types of injuries at national and subnational of Iran is limited. The evidence on the epidemiology of injuries in Iran are focused on the more prevalent types like RTI and falls and the available publications have used data from older national surveys which need to be updated to provide a holistic picture of injuries in country [[8], [9], [10]]. This paper aimed to assess the incidence of different injuries and associated behavioral risk factors such as non-compliance with helmets, seatbelts, and child safety seat use based upon findings of the Iran Stepwise approach to surveillance (STEPS) Survey 2021 [11], categorized by demographics and geographical region. As far as we know, this is the first study to investigate the geographic distribution of injuries in Iran.

## 2 Methods

### 2.1 Study design

The STEPS survey is a sequential large-scale cross-sectional population-based surveillance of NCD's risk factors. The current study was conducted using data from the eighth-round of the Iran STEPS survey which the survey protocol could be found elsewhere [11]. The process of collecting data based on the place of residence in a multistage clustered probability design and weighting adjustments were applied based on the Iran population and Housing Census 2016 [12]. Data were collected through face-to-face interviews. Around 10 % of participants were interviewed in early 2020 before the study was suspended due to the COVID-19 pandemic for a year, and the remaining 90 % of data collection occurred from February to April 2021 [11].

In this survey, adults aged 18 years or over from urban and rural areas of Iran's 31 provinces were examined for information on sociodemographic characteristics, health behaviors, history of metabolic risk factors, household assets, healthcare utilization, and anthropometric variables. All STEPS participants received detailed information about the study's objectives and methodology, and written consent was sought from all participants who agreed to participate.

### 2.2 Definition of variables

An injured individual was defined as someone who had been hurt in the preceding 12 months due to a road traffic accident, a cut, a fall, a burn, an animal attack, poisoning, drowning, self-harm, or interpersonal violence. This variable included the question: “Did you experience an accident within the past 12 months which led to the physical injury?”. Moreover, the injury “full recovery” and “partial recovery” are defined as being recovered in less than a month or one to six months, respectively. The study's independent variables included demographic features (sex, age, education, wealth index, and area of residence) and behavioral factors such as current smoking and drinking. Years of schooling were classified into four categories based on the number of successfully completed years of schooling [0 (Illiterate), 1–6, 7–12, and +12 years]. The participants' wealth index was calculated using analysis of household asset data and was classified into five quintiles, from the poorest (first quintile) to the wealthiest (fifth quintile). The body mass index (BMI) of each participant was calculated as weight (kg) divided by squared height (m2). Transportation-related risky behaviors consisted of the consumption of alcohol and smoking. Alcohol consumption included the question: “Have you consumed any alcoholic drinks in the past 12 months?” while heavy alcohol consumption was described as “at least six or more standard drinks in last month.”. Smoking included any usage of tobacco products like cigarettes or water pipes in the last 30 days, with heavy smoking defined as a “weekly number of cigarettes equal/more than 20”. Safety measures among car drivers and motorcyclists were defined as using a seatbelt in the front seats during the most recent road transportation and using a helmet during the last motorcycle ride, respectively. Moreover, child safety seat usage was evaluated by the answer to the question: “If you have a baby, did you use a child or baby car seat for the last time when using the car?”

### 2.3 Statistical analysis

Recruited data were utilized to offer descriptive statistics of variables of interest by subgroups of sex (male, female) and age (18–40, 41–65, 65+). In addition, the geographical data sampling frame allowed us to provide provincial-level findings. According to the 2016 National Population and Housing Census conducted by Iran's Statistical Center [12], age-standardization of the provincial incidence of injury was attained. All incidence was reported by mean and 95 % CIs.

To assess the association between the sociodemographic factors and the prevalence of safety measures among car drivers and motorcyclists, χ2 test was applied. A univariate and multiple logistic regression (Enter method) model was fitted to determine the association of each sociodemographic characteristic with RTI risk factor while adjusting for confounding effects. We used expert opinion to define variables for the model. The dichotomous variable for injuries factor was used as the dependent variable and sociodemographic variables as categorical independent covariates, while adjusted for other predictors in the model. A P-value of less than 0.05 was considered statistically significant. Data analyses were performed by R software version 4.0.5.

## 3 Results

The initial estimated sample size for the study consisted of 28821 individuals, distributed across 3176 clusters throughout the country. 27874 individuals who willingly provided informed consent were ultimately enrolled in the study of which 1538 (5.5 %, 95%CI: [5.2–5.8]) reported having been injured at least once in the past 12 months. The injured were, on average, 45.6 ± 0.5 years old and males accounted for 55.5 % of the cases. 22.2 % of the population were in the lowest wealth quantile, 18.4 % were in the highest wealth quantile, 60.6 % had basic insurance and 73.5 % were living in rural areas. In addition, 13.7 % of respondents had no formal education. 15.2 % of the injured population were over the age of 65. Of the respondents, 56.9 % reported full recovery from their injury within a month, while 38.0 % reported recovery taking longer than a month. Falls (44.4 %) were the most common cause of injury, followed by road traffic injury (21.7 %) and exposure to mechanical forces (16.5 %) (Table 1)

**Table 1:** Demographic and characteristics of participants with at least one incident injury in Iran in 2021.

	percent	n
Sex (percent [95%CI ])		
Male	55.5	873
Female	44.4	665
Age		
Mean (SD^a^)	Mean = 45.6	SD = 0.48
Age groups (percent [95%CI ])		
18-40	42.1	669
41-65	42.7	651
>65	15.2	218
Years of schooling (percent [95%CI ])		
Illiterate	13.7	216
1-6	23.7	365
7-12	19.8	307
12+	42.8	643
Wealth index ((percent [95%CI ])		
Q1^b^ (lowest)	22.2	334
Q2	21.1	311
Q3	20.0	289
Q4	18.2	259
Q5 (highest)	18.4	262
Insurance (percent [95%CI ])		
Not covered	9.6	153
Basic insurance	60.6	934
Complementary insurance	29.8	431
Residence (percent [95%CI ])		
Urban	26.5	432
Rural	73.5	1106
Obesity (percent [95%CI ])		
Underweight (BMI^c^ < 18.5 kg/m2)	3.2	51
Normal (BMI 18.5–24.9 kg/m2)	33.9	525
Overweight (BMI 25.0–29.9 kg/m2)	38.3	590
Obese (BMI≥30.0 kg/m2)	24.6	366
Injury type (percent [95%CI ])		
Traffic	21.7	350
Burn	2.1	29
Falling	44.4	663
Drown	0.2	2
Shock	0.2	3
Poison	0.7	9
Suicide	0.3	4
Violence	1.6	22
Scorpion or snake bite	0.2	3
Animal attack	0.8	14
Exposure to mechanical forces	16.5	256
Other	11.2	183
Injury recovery (percent [95%CI ])		
Fully recovered	56.9	869
Partially recovered	38.0	589
No recovery	5.1	80

a SD: Standard deviation.

b Q: Quintile.

c BMI: Body Mass Index.

Except for falls and burns, males had a higher proportion of all types of injuries, as shown in Table 2. Fall's greatest prevalence was seen in females of the 41–65 age group (52.5 %, 95 % CI: [46.7–58.2]), followed by the >65 age group (25.9 % [20.8–30.9]). Moreover, disaggregated by age group, people more than 65 years old accounted for 6.4 % (3.0–9.9) of the male RTI cases, and 9.0 % (2.42–15.57) of female cases. Injuries due to suicide were only reported in the 18–40 age group and distributed equally between males and females. Drowning, electric shock, scorpion, and snake bite were incidents that were only observed in males, according to Table 2.

**Table 2:** Age and sex distribution of injuries.

Injury	Females			Males		
	18–40	41–65	>65	18–40	41–65	>65
Road traffic (estimate [95 % CI)	40.4 (29.9, 51.0)	50.6 (39.7, 61.4)	9.0 (2.4, 15.6)	57.2 (50.2, 64.1)	36.4 (29.6, 43.2)	6.4 (3.0, 9.9)
Burn	69.5 (44.8, 94.3)	14.0 (−4.9, 32.9)	16.43 (−3.0, 35.9)	72.73 (48.3, 97.2)	27.3 (2.8, 51.7)	-a
Falling	21.6 (17.0, 26.2)	52.5 (46.7, 58.2)	25.9 (20.8, 30.9)	39.3 (33.0, 45.6)	39.0 (32.6, 45.3)	21.7 (15.8, 27.6)
Drown	–	–	–	–	100 (100, 100)	–
Shock	–	–	–	39.8 (−18.8, 98.4)	60.2 (1.6, 118.8)	–
Poison	72.4 (28.1, 116.7)	27.6 (−16.7, 71.9)	–	64.3 (10.9, 117.8)	35.7 (−17.7, 89.1)	–
Suicide	100 (100, 100)	–	–	100 (100, 100)	–	–
Violence	46.6 (9.4, 83.9)	18.7 (−9.1, 46.6)	34.6 (−2.7, 72.0)	75.6 (51.1, 100.2)	24.4 (−0.2, 48.9)	–
Scorpion or snake bite	–	–	–	14.4 (−15.7, 44.5)	85.6 (55.5, 115.7)	–
Animal attack	38.9 (6.2, 86.0)	53.4 (12.3, 90.4)	7.7 (0.9, 44.3)	41.7 (14.3, 75.4)	22.9 (6.4, 56.2)	35.4 (9.9, 73.3)
Hit	39.6 (28.5, 51.8)	43.04 (31.62, 55.25)	17.4 (9.78, 29.03)	52.42 (44.15, 60.57)	39.6 (31.83, 47.94)	7.98 (4.55, 13.61)
Other	49.5 (37.3, 61.1)	43.2 (31.1, 55.3)	7.2 (1.5, 13.0)	57.3 (46.4, 68.1)	39.7 (29.0, 50.5)	3.0 (−1.1, 7.1)

Data are presented as percentage and 95 % CI.

a Inadequate data.

As Table 3 highlights, 90.0% of all respondents reported using seat belts the last time they were the driver or the front-seat passenger. Males reported higher seatbelt use than females while sitting on front seats, 93.0 % (92.4–93.7) versus 87.3 % (86.5–88.0), respectively. Moreover, only 34.4 % (33.6–35.2) of the back seat passengers said they used seat belts the last time they were in a car. Only 9.4 % (8.9–10.0) of the participants used safety car seats for their children and males are significantly more likely to use them than females (P < 0.001). Prevalence of helmet usage was 18.6 % (17.7–19.6), with the highest rates among males (27.0 % [25.6–28.4]) than females (5.6 % [4.7–6.5]). In addition, males were more likely to use a car for intercity (72.2 % [71.3–73.0]) or countryside (59.4 % [58.5–60.4]) travel than females (71.6 % [70.8–72.4] and 53.1 % [52.3–54.0], respectively).

**Table 3:** Safety measures among car drivers and motorcyclists.

	Total	Males	Females	P-value
Car drive				
• Seatbelt (front seat)	90.0 (89.5, 90.5)	93.0 (92.4, 93.7)	87.3 (86.5, 88.1)	<0.001
• Seatbelt (rear seat)	34.4 (33.6, 35.2)	31.9 (30.8, 33.1)	36.6 (35.4, 37.7)	<0.001
• Baby seat	9.4 (8.9, 10.0)	10.7 (9.8, 11.5)	8.4 (7.7, 9.2)	0.0004
• Driving in countryside	55.9 (55.3, 56.6)	59.4 (58.5, 60.4)	53.1 (52.3, 54.0)	<0.001
• Driving in intercity highways	71.8 (71.2, 72.4)	72.2 (71.3, 73.1)	71.6 (70.8, 72.4)	0.3147
Motorcycle ride				
• Helmet use	18.6 (17.7, 19.6)	27.0 (25.6, 28.4)	5.60 (4.7, 6.5)	<0.001
• Full-fledge	67.1 (64.4, 69.8)	67.4 (64.5, 70.3)	65.3 (57.2, 73.4)	<0.001
• Tricot	25.9 (23.3, 28.4)	25.6 (22.9, 28.3)	28.2 (20.5, 35.8)	<0.001
• Other types	6.9 (5.5, 8.4)	7.0 (5.5, 8.6)	6.5 (2.3, 10.6)	<0.001

-Data are presented as percentage and 95 % CI.

-P< 0.05 was considered as statistically significant.

The logistic regression analysis showed that RTI was higher among males (OR: 1.7, [1.5–2.0]) compared to females based on the age-adjusted OR (Table 4). There was no significant statistical association between age, BMI, education, type of insurance, and having RTI. Being in the richest quintile was a protective factor (OR: 0.8 [0.6–1.0]) while being an occasional or heavy alcohol drinker (OR: 2.0 [1.3–3.0] and OR: 2.7 [1.7–4.1], respectively) was a significant behavioral risk factor associated with RTIs. Urban inhabitants showed no difference in risk of RTI compared to rural residents (OR: 1.0 [0.8–1.2]). There was no significant statistical association between using seatbelt in the intercity drive and having RTI (OR: 1.0 [0.8,1.2] for front seat, OR: 1.1 [0.9,1.4] for back seat passengers)

**Table 4:** Logistic regression of road traffic injuries factors.

	Univariable		Adjusted	
	OR***(95 % CI)	P-value	OR (95 % CI)	P-value
Sex				
Female [ref]		–		
Male	1.6 (1.4,1.8)	<0.001	1.7 (1.5, 2.0)	<0.001
Age				
age	1 (0.9,1.0)	0.927	0.9 (0.9,1.0)	0.012
Education				
Illiterate [ref]	–	–		
1-7	0.9 (0.8,1.2)	0.794	0.9 (0.7,1.2)	0.666
7-12	1.0 (0.8,1.2)	0.848	0.9 (0.7,1.2)	0.519
12+	0.9 (0.8,1.1)	0.669	1.0 (0.8,1.4)	0.766
Wealth				
Q1* [ref]	–	–		
Q2	0.9 (0.7,1.1)	0.292	0.9 (0.7,1.1)	0.363
Q3	0.9 (0.8,1.1)	0.412	0.9 (0.7,1.1)	0.205
Q4	0.8 (0.7,1.0)	0.063	0.9 (0.7,1.1)	0.266
Q5	0.8 (0.7,0.9)	0.018	0.8 (0.6,1.0)	0.039
BMI				
BMI**	1.0 (0.9,1.0)	0.555	1.0 (0.9,1.0)	0.17
Residence				
Urban [ref]	–	–		
Rural	0.9 (0.8,1.0)	0.209	0.99 (0.8,1.2)	0.958
Alcohol consumption				
No [ref]	–	–		
Occasional	2.6 (1.7,3.8)	<0.001	2.0 (1.3,3.0)	0.001
Heavy	3.6 (2.4,5.4)	<0.001	2.7 (1.7,4.1)	<0.001
Smoking habit				
No [ref]	–	–		
Occasional	1.8 (1.4,2.2)	<0.001	1.2 (0.9,1.6)	0.311
Heavy	1.7 (1.4,2.1)	<0.001	1.1 (0.8,1.3)	0.653
Safety measures				
Seatbelt (front seat)	0.8 (0.7,0.9)	0.004	1.0 (0.8,1.2)	0.934
Seatbelt (rear seat)	0.9 (0.7,1.0)	0.06	1.1 (0.9,1.4)	0.266

*Q: Quintile; **BMI: Body Mass Index,*** *Multivariate Logistic Regression using Enter Method.

-Data are presented percentage and 95 % CI.

-P< 0.05 was considered as statistically significant.

**Fig. 1:**
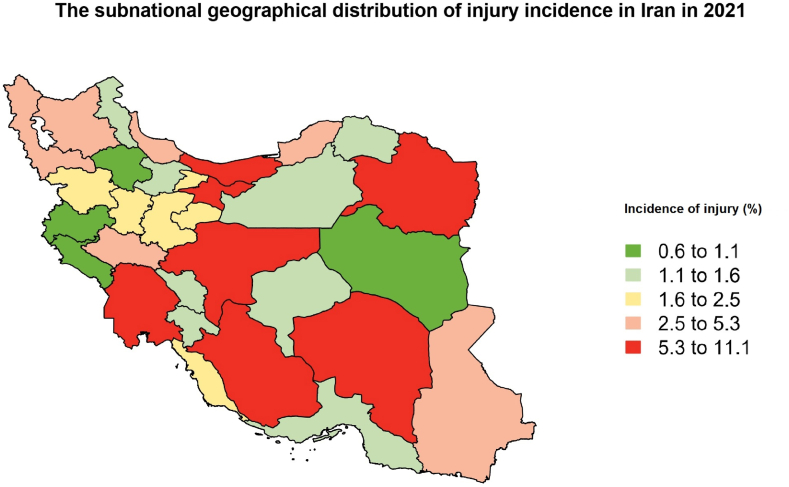
The subnational geographical distribution of injury incidence in Iran in 2021.

The highest incidence of injury in males was observed in Razavi Khorasan (11.2 % [8.9–14.0]) and Isfahan (10.9 % [8.7–13.6]), as shown in Appendix Table 1. In contrast to Razavi Khorasan, South Khorasan (0.7 % [0.5–1.2]) had the lowest injury rates of all the provinces. Injury incidence among females varied by nearly 11% points in the best-off and worst-off provinces in Ilam (0.2 % [0.1–0.6]) and Tehran (12.0 % [9.3–15.2]). The subnational geographical distribution of injury incidence in Iran has shown in Fig. 1. Animal attack was only observed in 12 provinces and mainly upon males. Similarly, scorpion or snake bite was reported exclusively in males in Khorasan Razavi, Kurdistan, and North Khorasan. For additional information on the provincial distribution of injuries, visit Appendix Table 2.

## 4 Discussion

The current population-based survey enrolling more than 27,500 adults over 18 years represented the first national-wide evaluation of the epidemiology of injuries and associated factors in Iran at both national and provincial levels. Our analysis of STEPS 2021 data showed the crude incidence of injured adult individuals to be 55 per 1000 people in the past 12 months. The leading cause of injuries in this study was falls, followed by RTIs and exposure to mechanical forces. Males, heavy smokers, and moderate or heavy alcohol drinkers were significantly more likely to be injured in a road traffic crash. Additionally, the incidence of different injuries showed an uneven distribution in the provinces of Iran. Although injury prevalence has been a major health concern in many developing countries, the predisposing factors and associated health outcomes differ by population. The injury incidence in our study was lower than what has been reported in countries such as Germany [13], Kenya [14], and Thailand [15]. This lower incidence may be attributed to the lockdown due to the COVID-19 pandemic that led to a marked decrease in the rate of injuries worldwide [16].

Falls were the leading cause of injury according to this study, which is consistent with previous data reported from Iran [17,18]. Also, in the 5–44 year age group, fall injuries were the leading cause of years of life lost [18]. This study showed a higher incidence of falls among females older than 45. *Saadat* et al. [10]*,* in a survey of fall-related injuries in Iran, showed that age between 45 and 64 was associated with higher incidence and severity of fall events. Sex disparity in the distribution of nonfatal fall injuries has been well documented [19,20]. The heightened incidence of fall injuries among women, in comparison to men, can be attributed to distinction in muscle strength exists between genders and the higher prevalence of osteoporosis among women [21]. According to the GBD estimates in 2019, falls were the 21st leading cause of disability-adjusted life years, while it ranked 8th among females in the oldest age group (+75 years) [1]. In the elderly, a minor fall can lead to long-term hospitalization and lifelong impairment [22]. Nevertheless, injuries due to falling in this age group require attention, as injuries have far-reaching consequences for both individuals and healthcare systems [23]. Several contributors to fall injuries in the elderly have been suggested including sedative drugs, reduced balance, depression, and cognitive impairment [24,25]. Evidence supports the effectiveness of multifactorial interventions such as physical exercise and home safety modifications [26,27]. A greater understanding of the factors that contribute to falls in Iran might allow the development of prevention programs.

Suicide injury was only reported in the 18–40 age group and proportionally borne by males and females. Suicide rates in Iran were reported to be significantly higher in the 26–40 age group and male sex [28]. Suicide showed to be responsible for 3.9 % of emergency department admissions in Iran [29]. However, our data were probably underreported because such a survey may not capture them accurately due to stigma related to Iranian society's culture and religion [30]. Moreover, we have only assessed unsuccessful suicide cases and mortality has not been included in our statistics; therefore, this may result in a selection bias and underestimation of injury rates due to this cause.

This study has found that certain groups of people are more vulnerable to traffic injuries than others. Similar to most studies in Iran and other counteries [31,32], we found a significantly higher RTI incidence among males compared to females. Other studies suggested it be attributed to males taking riskier behaviors, but rather than males spending more time driving than females. Moreover, socioeconomic status has been shown to be a significant predictor of RTIs in this study, as those with the highest incomes were at a decreased risk of RTIs occurrence. There have been other studies that have shown comparable results [33,34]. Contrary to what we expected, urban inhabitants showed no difference in risk of road injuries compared to rural residents, which is in contrast to previous studies conducted in Iran [35,36]. This may be a result of recent policies regarding public education and awareness, the development of road structures, and national speed limit law [9,37]. Installation of speed cameras, for example, has been shown to be quite effective in reducing over-speeding in the proximity of cameras [38,39].

In terms of RTI mortality rates, Iran is ranked third among higher-middle-income countries in the world [38]. As a major public health issue, deaths due to RTI have increased from 12.64 to 29.1 per 100,000 people during 1990–2015 [9]. In this regard, the Iranian Non-Communicable Diseases Committee (INCDC) established a national action plan for NCD Prevention and Control in 2015, with a stated goal of a 20% relative decrease in the mortality rate caused by road injuries by 2025 [40]. Despite mandatory laws in Iran for the use of seatbelts and helmets, risky behaviors such as speeding and failure to use helmets, seatbelts, and car seats for children continue to be featured among the Iranian population nationwide [5]. The national front-seat seatbelt and helmet compliance rates were found to be 90.0 % and 18.6 %, respectively, which is comparable with previous data reported from Iran [8]. The use of child car seats was only reported in 9.4 % of the participants, which is higher than Moradi et al. [41] report as a prevalence of 4.3 %, but aligns with the higher use of child seats among female drivers. In Iran the use of safety equipment, including child car seats, have been advised but lacks mandatory laws.

This study examined the association between RTIs and smoking, an area with a lack of data. Not only are smokers at risk for chronic diseases such as cancer and lung disease, but they also have a higher risk of RTIs than non-smokers [42]. Tobacco use may contribute to vehicular trauma through factors like driving distractions, the effects of carbon monoxide on driver's alertness, and cognitive impairments due to prolonged nicotine use [43,44]. Moreover, smokers are more likely to engage in risky behaviors [43]. Being a heavy smoker or moderate to heavy alcohol drinker was a strong behavioral determinant of RTIs in this study. Although the consumption of alcohol is legally prohibited in Iran, the 24–34 age group intended to be the main consumers [45]. Previous research has stated that alcohol consumption is the leading risk factor for RTIs in this country [6]. Additionally, alcohol-impaired driving has been associated with seatbelt use non-compliance [8].

We compared sex-specific incidence rates of injuries in 31 provinces of Iran. The highest provincial incidence of injury in males (Razavi Khorasan) was almost fifteen times higher than the lowest incidence (South Khorasan). Drowning was reported in Mazandaran and Isfahan provinces exclusively. Mazandaran is one of the provinces with the most access to seawater and the highest mortality rate from drowning annually [46]. Razavi Khorasan, Kurdistan, and North Khorasan were the provinces with reports of scorpion or snake bites in males. A seven-year survey in Razavi Khorasan estimated the annual incidence of snakebite and scorpion sting to be 1.3 and 0.5 per 100,000 respectively in this province [47]. Iran, along with Mexico, has the highest rate of scorpion stings, which is more common in particular provinces [48].

Our data showed that the overall incidence of injuries in Iran's young adult (18–40 years) population is high. Injuries are a substantial contributor to expensive medical costs, poor mental health, and lost productivity, in addition to their immediate health consequences [3]. An analysis of health care performance in Iran showed an increased rate of years lived with disability component of the disability-adjusted life years (DALYs) due to injuries in recent years [40,49]. Injuries affect not just the afflicted individual but also his or her family, friends, and communities and are a significant economic burden on nations [50]. The lack of nationally representative data highlights the importance of investing in the registration of injury incidents and causes. These data are also necessary for determining and monitoring the efficacy of injury intervention strategies.

## 5 Limitations, strengths, and future direction

To the best of our knowledge, this is the first examination of injuries at the national and provincial levels of Iran, which provides an excellent insight into the inequality in the distribution of injuries across the country and between males and females, and may provide information for national and regional policymakers and health care providers for targeted preventive strategies. In order to address the country's burden of injury, it is essential to continue to monitor and carry out further research.

With regards to the limitations of this survey, one was that the statistics were derived from self-reported information through interviews, which is vulnerable to recall bias. Participants may have forgotten the incidence in the 12-month period considered to recall being injured. We attempted to minimize this bias by carefully structuring our survey questions. A clinical damage severity assessment was not possible in this survey, and no data on hospitalization or mortality rate were collected. However, while the majority of studies on injury epidemiology are conducted in hospitals and concentrate on RTIs incidence and severity, this population-based study evaluated all kinds of intentional and unintentional injuries to obtain a clear image of injury patterns in Iran. Moreover, we cannot ascertain the impacts of the lockdown due to the COVID-19 pandemic on rates of different kinds of injuries. Therefore, future investigation should be directed toward the potential effects of the COVID-19 pandemic lockdown considering factors such as changes in daily activities, mobility patterns, and healthcare accessibility. Additionally, an in-depth analysis of the impact of recent policies aimed at reducing road traffic injuries could provide valuable insights for designing effective injury prevention strategies and public health interventions.

## 6 Conclusion

Our study highlighted that injuries are a common health problem in Iran, with uneven distribution among provinces. Our sex-specific data established a clear image of injury patterns across the country. Males, especially at young ages, accounted for a great number of injury cases and this target group inquired further investigation in terms of preventive intervention. This epidemiological data have several implications for healthcare practice, policy, and future research. Public health authorities and policymakers can target areas with a greater incidence of specific injury types to modify safety interventions if they have a better understanding of geographic variation. This investigation addresses some knowledge gaps regarding factors related to RTIs and provides insights that could be used for RTI prevention interventions in Iran and other low- and middle-income countries.

## Data availability

Data associated with this study haven't been deposited into a publicly available repository. All data used in this manuscript are available upon an official inquiry to the corresponding author.

## Role of funder

This work did not receive any financial support from any organization.

## Ethics statement

All participants of the STEPS Survey 2021 provided an informed consent before participation and the study protocol was approved by the ethical committee of the National Institute for Health Research (ID: IR. TUMS.NIHR.REC.1398.006). In this work, as secondary unidentified data was analyzed and reported, the institution waived the need for further approval.

## CRediT authorship contribution statement

**Elnaz Shahmohamadi:** Writing – review & editing, Writing – original draft, Formal analysis. **Erfan Ghasemi:** Visualization, Validation, Methodology, Formal analysis. **Esmaeil Mohammadi:** Writing – original draft, Methodology. **Maryam Nasserinejad:** Formal analysis. **Sina Azadnajafabad:** Writing – review & editing. **Mohammad-Reza Malekpour:** Writing – review & editing, Data curation. **Mohammad-Mahdi Rashidi:** Writing – review & editing, Supervision, Data curation. **Naser Ahmadi:** Supervision, Formal analysis. **Negar Rezaei:** Project administration, Conceptualization. **Mohammadreza Naderian:** Writing – review & editing. **Moein Yoosefi:** Visualization, Formal analysis. **Yosef Farzi:** Resources, Investigation. **Nazila Rezaei:** Writing – review & editing, Conceptualization. **Rosa Haghshenas:** Resources, Project administration, Amirali Hajebi, Resources, Project administration. **Elham Abdolhamidi:** Resources, Investigation, Data curation. **Ali Golestani:** Writing – review & editing. **Ameneh Kazemi:** Data curation. **Mahdi Delaram Dizaj:** Project administration, Data curation. **Niusha Nazari:** Investigation. **Azadeh Momen Nia Rankohi:** Resources. **Mahbobeh Darman:** Supervision, Resources, Project administration. **Shirin Djalalinia:** Project administration, Methodology. **Alireza Moghisi:** Writing – review & editing. **Farshad Farzadfar:** Writing – review & editing, Validation, Supervision, Resources, Project administration.

## Declaration of competing interest

The authors declare that they have no known competing financial interests or personal relationships that could have appeared to influence the work reported in this paper.
