# Supplementary material for: “Current incidence of injuries in Iran; findings of STEPS survey 2021”
Source: Heliyon. 2023 Oct 14;9(11):e20907. doi: 10.1016/j.heliyon.2023.e20907 (PMC10618784; doi:10.1016/j.heliyon.2023.e20907)
Supplement: Multimedia component 1 [file mmc1.docx]

#

# Appendix table 1. The provincial composition of injury in Iran in 2021

The prevalence of injury stratified by sex among provinces. (All values are provided with 95% uncertainty interval (95% UI))

| **Province** | **Sex** | **Percent (CI)** |
| --- | --- | --- |
| Alborz | Female | 2.35(1.31-4.18) |
|  | Male | 1.56(0.81-2.95) |
| Ardabil | Female | 0.99(0.51-1.88) |
|  | Male | 1.73(1.11-2.7) |
| Azerbaijan, East | Female | 4.33(2.82-6.58) |
|  | Male | 4.27(2.89-6.26) |
| Azerbaijan, West | Female | 4.4(2.87-6.68) |
|  | Male | 4.17(2.83-6.11) |
| Bushehr | Female | 1.7(1.2-2.4) |
|  | Male | 1.59(1.15-2.19) |
| Chahar Mahaal and Bakhtiari | Female | 1.03(0.66-1.61) |
|  | Male | 1.12(0.77-1.65) |
| Fars | Female | 5.7(3.93-8.2) |
|  | Male | 7.05(5.25-9.41) |
| Gilan | Female | 3.67(2.27-5.9) |
|  | Male | 1.78(0.96-3.26) |
| Golestan | Female | 2.98(2.06-4.27) |
|  | Male | 2.72(1.93-3.81) |
| Hamadan | Female | 2.74(1.84-4.05) |
|  | Male | 2.2(1.48-3.26) |
| Hormozgan | Female | 1.52(0.82-2.79) |
|  | Male | 1.69(1.01-2.82) |
| Ilam | Female | 0.22(0.08-0.57) |
|  | Male | 0.98(0.65-1.49) |
| Isfahan | Female | 8.42(6.27-11.21) |
|  | Male | 10.93(8.73-13.61) |
| Kerman | Female | 6.39(4.51-8.98) |
|  | Male | 4.96(3.47-7.05) |
| Kermanshah | Female | 0.76(0.36-1.6) |
|  | Male | 1.05(0.6-1.83) |
| Khorasan, North | Female | 0.77(0.45-1.29) |
|  | Male | 1.33(0.93-1.89) |
| Khorasan, Razavi | Female | 8.5(6.28-11.41) |
|  | Male | 11.25(8.95-14.05) |
| Khorasan, South | Female | 0.71(0.41-1.22) |
|  | Male | 0.74(0.46-1.19) |
| Khuzestan | Female | 7.11(4.79-10.45) |
|  | Male | 6.97(4.96-9.72) |
| Kohgiluyeh and Boyer-Ahmad | Female | 0.96(0.6-1.52) |
|  | Male | 1.63(1.19-2.23) |
| Kurdistan | Female | 2.73(1.85-4.02) |
|  | Male | 1.95(1.29-2.94) |
| Lorestan | Female | 3.4(2.39-4.83) |
|  | Male | 2(1.32-3.02) |
| Markazi | Female | 1.44(0.84-2.47) |
|  | Male | 1.93(1.28-2.9) |
| Mazandaran | Female | 6.71(4.77-9.38) |
|  | Male | 4.21(2.85-6.17) |
| Qazvin | Female | 1.42(0.83-2.42) |
|  | Male | 1.47(0.92-2.35) |
| Qom | Female | 2.13(1.38-3.26) |
|  | Male | 1.63(1.04-2.53) |
| Semnan | Female | 1.08(0.7-1.67) |
|  | Male | 1.08(0.73-1.59) |
| Sistan and Baluchistan | Female | 1.93(1.2-3.1) |
|  | Male | 2.98(2.13-4.16) |
| Tehran | Female | 11.9(9.29-15.12) |
|  | Male | 10.38(8.16-13.11) |
| Yazd | Female | 1.03(0.64-1.63) |
|  | Male | 1.56(1.12-2.16) |
| Zanjan | Female | 1(0.63-1.59) |
|  | Male | 1.1(0.75-1.63) |

# Appendix table 2. The national and provincial composition of different injuries in Iran in 2021

The prevalence of each injury stratified by sex among provinces proportionally to the weight of population in each province. (All values are provided with 95% uncertainty interval (95% UI))

|  | **Province** | | **Sex** | **Percent** | **Lower percentage** | **Upper percentage** |
| --- | --- | --- | --- | --- | --- | --- |
| Animal attack | | Azerbaijan, East | Female | 30.11 | 4.11 | 81.23 |
|  |  | Fars | Female | 38.87 | 6.16 | 86.02 |
|  |  |  | Male | 18.5 | 2.69 | 65.08 |
|  |  | Hamadan | Male | 9.48 | 1.26 | 46.27 |
|  |  | Ilam | Male | 4.58 | 0.58 | 28.14 |
|  |  | Isfahan | Male | 17.71 | 2.55 | 63.89 |
|  |  | Khorasan, North | Female | 7.79 | 0.88 | 44.57 |
|  |  | Khorasan, South | Female | 7.7 | 0.87 | 44.26 |
|  |  | Lorestan | Male | 9.2 | 1.22 | 45.46 |
|  |  | Markazi | Male | 9.28 | 1.23 | 45.7 |
|  |  | Mazandaran | Male | 17.68 | 2.55 | 63.84 |
|  |  | Qazvin | Female | 15.53 | 1.84 | 64.33 |
|  |  |  | Male | 9.15 | 1.21 | 45.31 |
|  |  | Semnan | Male | 4.42 | 0.56 | 27.4 |
| Burn | | Azerbaijan, East | Male | 8.99 | 1.26 | 43.32 |
|  |  | Azerbaijan, West | Female | 18.62 | 4.83 | 50.78 |
|  |  | Chahar Mahaal and Bakhtiari | Female | 2.21 | 0.29 | 14.78 |
|  |  | Fars | Male | 9.1 | 1.28 | 43.66 |
|  |  | Golestan | Female | 5.01 | 0.68 | 28.88 |
|  |  | Isfahan | Female | 18.12 | 4.68 | 49.94 |
|  |  | Kerman | Male | 8.61 | 1.2 | 42.18 |
|  |  | Khorasan, North | Male | 2.56 | 0.34 | 16.83 |
|  |  | Khorasan, Razavi | Female | 18.85 | 4.9 | 51.15 |
|  |  |  | Male | 18.34 | 4.7 | 50.53 |
|  |  | Kohgiluyeh and Boyer-Ahmad | Female | 2.32 | 0.31 | 15.42 |
|  |  |  | Male | 2.23 | 0.3 | 14.97 |
|  |  | Kurdistan | Female | 6.87 | 0.95 | 36.22 |
|  |  | Lorestan | Female | 4.73 | 0.64 | 27.64 |
|  |  | Markazi | Male | 9.14 | 2.17 | 31.3 |
|  |  | Mazandaran | Female | 8.99 | 1.27 | 43.22 |
|  |  |  | Male | 18.42 | 4.73 | 50.68 |
|  |  | Qazvin | Female | 4.62 | 0.63 | 27.16 |
|  |  | Sistan and Baluchistan | Female | 9.66 | 2.32 | 32.49 |
|  |  |  | Male | 4.38 | 0.59 | 26.16 |
|  |  | Tehran | Male | 18.23 | 4.67 | 50.36 |
| Drown | | Isfahan | Male | 50.7 | 6.03 | 94.28 |
|  |  | Mazandaran | Male | 49.3 | 5.72 | 93.97 |
| Falling | | Alborz | Male | 0.48 | 0.07 | 3.33 |
|  |  |  | Female | 2.68 | 1.29 | 5.5 |
|  |  | Ardabil | Female | 0.6 | 0.19 | 1.85 |
|  |  |  | Male | 2.72 | 1.46 | 5 |
|  |  | Azerbaijan, East | Female | 3.43 | 1.8 | 6.45 |
|  |  |  | Male | 4.97 | 2.7 | 8.95 |
|  |  | Azerbaijan, West | Female | 3.57 | 1.87 | 6.7 |
|  |  |  | Male | 4.07 | 2.06 | 7.9 |
|  |  | Bushehr | Male | 1.08 | 0.56 | 2.08 |
|  |  |  | Female | 1.41 | 0.84 | 2.34 |
|  |  | Chahar Mahaal and Bakhtiari | Male | 0.63 | 0.26 | 1.51 |
|  |  |  | Female | 0.77 | 0.38 | 1.54 |
|  |  | Fars | Female | 6.51 | 4.11 | 10.18 |
|  |  |  | Male | 6.61 | 3.89 | 11.02 |
|  |  | Gilan | Male | 0.99 | 0.25 | 3.89 |
|  |  |  | Female | 4.17 | 2.26 | 7.58 |
|  |  | Golestan | Male | 2.47 | 1.33 | 4.54 |
|  |  |  | Female | 3.55 | 2.27 | 5.51 |
|  |  | Hamadan | Male | 1.57 | 0.7 | 3.46 |
|  |  |  | Female | 2.93 | 1.77 | 4.81 |
|  |  | Hormozgan | Female | 0.81 | 0.26 | 2.47 |
|  |  |  | Male | 2.13 | 0.96 | 4.66 |
|  |  | Ilam | Female | 0.19 | 0.05 | 0.76 |
|  |  |  | Male | 0.51 | 0.19 | 1.36 |
|  |  | Isfahan | Female | 7.76 | 5.13 | 11.56 |
|  |  |  | Male | 13.05 | 9.15 | 18.27 |
|  |  | Kerman | Male | 5.84 | 3.36 | 9.95 |
|  |  |  | Female | 7.37 | 4.78 | 11.2 |
|  |  | Kermanshah | Female | 0.97 | 0.41 | 2.32 |
|  |  |  | Male | 1.25 | 0.52 | 2.97 |
|  |  | Khorasan, North | Female | 0.77 | 0.39 | 1.55 |
|  |  |  | Male | 1.63 | 0.94 | 2.81 |
|  |  | Khorasan, Razavi | Female | 8.28 | 5.48 | 12.31 |
|  |  |  | Male | 13.02 | 9.01 | 18.46 |
|  |  | Khorasan, South | Female | 0.67 | 0.32 | 1.4 |
|  |  |  | Male | 0.77 | 0.34 | 1.71 |
|  |  | Khuzestan | Male | 5.09 | 2.08 | 11.95 |
|  |  |  | Female | 6.47 | 3.45 | 11.78 |
|  |  | Kohgiluyeh and Boyer-Ahmad | Female | 0.94 | 0.51 | 1.75 |
|  |  |  | Male | 1.84 | 1.1 | 3.05 |
|  |  | Kurdistan | Male | 2.1 | 1.04 | 4.18 |
|  |  |  | Female | 2.5 | 1.45 | 4.28 |
|  |  | Lorestan | Male | 2.27 | 1.18 | 4.32 |
|  |  |  | Female | 3.42 | 2.13 | 5.45 |
|  |  | Markazi | Female | 1.2 | 0.54 | 2.66 |
|  |  |  | Male | 1.76 | 0.84 | 3.66 |
|  |  | Mazandaran | Male | 5.11 | 2.78 | 9.22 |
|  |  |  | Female | 7.8 | 5.12 | 11.72 |
|  |  | Qazvin | Female | 0.99 | 0.41 | 2.37 |
|  |  |  | Male | 1.52 | 0.68 | 3.34 |
|  |  | Qom | Male | 1.76 | 0.84 | 3.66 |
|  |  |  | Female | 1.87 | 1.01 | 3.44 |
|  |  | Semnan | Male | 0.5 | 0.19 | 1.35 |
|  |  |  | Female | 0.95 | 0.51 | 1.76 |
|  |  | Sistan and Baluchistan | Male | 1.55 | 0.69 | 3.42 |
|  |  |  | Female | 1.6 | 0.8 | 3.19 |
|  |  | Tehran | Male | 9.66 | 6.26 | 14.6 |
|  |  |  | Female | 13.41 | 9.82 | 18.04 |
|  |  | Yazd | Female | 1.23 | 0.69 | 2.18 |
|  |  |  | Male | 1.91 | 1.14 | 3.18 |
|  |  | Zanjan | Male | 1.13 | 0.59 | 2.18 |
|  |  |  | Female | 1.18 | 0.66 | 2.07 |
| Violence | | Azerbaijan, East | Male | 14.25 | 2.08 | 56.48 |
|  |  | Azerbaijan, West | Female | 30.11 | 7.65 | 69.14 |
|  |  |  | Male | 19.08 | 4.83 | 52.28 |
|  |  | Fars | Male | 19.29 | 4.89 | 52.62 |
|  |  | Gilan | Male | 9.59 | 1.34 | 45.41 |
|  |  | Hamadan | Male | 5.01 | 0.67 | 29.2 |
|  |  | Khorasan, North | Male | 5.17 | 1.18 | 19.99 |
|  |  | Khorasan, Razavi | Female | 19.66 | 2.86 | 67.08 |
|  |  | Khuzestan | Female | 31.65 | 8.17 | 70.67 |
|  |  |  | Male | 20.49 | 5.25 | 54.5 |
|  |  | Kohgiluyeh and Boyer-Ahmad | Male | 4.72 | 1.07 | 18.46 |
|  |  | Qom | Female | 14.89 | 3.33 | 47.03 |
|  |  | Semnan | Female | 3.69 | 0.46 | 23.99 |
|  |  |  | Male | 2.41 | 0.32 | 16.14 |
| other | | Alborz | Female | 1.85 | 0.26 | 11.96 |
|  |  |  | Male | 3.22 | 0.81 | 11.94 |
|  |  | Ardabil | Female | 1.83 | 0.45 | 7.1 |
|  |  |  | Male | 3.15 | 1.18 | 8.13 |
|  |  | Azerbaijan, East | Female | 5.87 | 1.92 | 16.59 |
|  |  |  | Male | 3.31 | 0.83 | 12.26 |
|  |  | Azerbaijan, West | Female | 7.74 | 2.96 | 18.75 |
|  |  |  | Male | 1.76 | 0.25 | 11.42 |
|  |  | Bushehr | Female | 2.79 | 1.23 | 6.22 |
|  |  |  | Male | 2.4 | 1.06 | 5.36 |
|  |  | Chahar Mahaal and Bakhtiari | Female | 1.89 | 0.69 | 5.02 |
|  |  |  | Male | 1.93 | 0.79 | 4.64 |
|  |  | Fars | Female | 5.9 | 1.93 | 16.68 |
|  |  |  | Male | 3.16 | 0.79 | 11.72 |
|  |  | Gilan | Female | 5.65 | 1.85 | 16.01 |
|  |  | Golestan | Female | 0.92 | 0.13 | 6.29 |
|  |  |  | Male | 5.35 | 2.54 | 10.9 |
|  |  | Hamadan | Female | 5.38 | 2.22 | 12.46 |
|  |  |  | Male | 0.82 | 0.11 | 5.58 |
|  |  | Hormozgan | Female | 1.33 | 0.19 | 8.89 |
|  |  |  | Male | 2.19 | 0.55 | 8.34 |
|  |  | Ilam | Male | 1.22 | 0.39 | 3.76 |
|  |  | Isfahan | Female | 1.83 | 0.26 | 11.87 |
|  |  |  | Male | 10.59 | 5.18 | 20.44 |
|  |  | Kerman | Female | 3.87 | 0.97 | 14.17 |
|  |  |  | Male | 3.09 | 0.78 | 11.49 |
|  |  | Kermanshah | Female | 0.97 | 0.13 | 6.58 |
|  |  | Khorasan, North | Male | 1.2 | 0.38 | 3.69 |
|  |  | Khorasan, Razavi | Female | 15.36 | 7.97 | 27.54 |
|  |  |  | Male | 6.35 | 2.42 | 15.63 |
|  |  | Khorasan, South | Female | 1.48 | 0.47 | 4.57 |
|  |  | Khuzestan | Female | 7.73 | 2.95 | 18.76 |
|  |  |  | Male | 9.9 | 4.55 | 20.19 |
|  |  | Kohgiluyeh and Boyer-Ahmad | Male | 1.96 | 0.81 | 4.71 |
|  |  | Kurdistan | Female | 6.51 | 3.09 | 13.2 |
|  |  |  | Male | 2.41 | 0.77 | 7.27 |
|  |  | Lorestan | Female | 0.97 | 0.14 | 6.59 |
|  |  |  | Male | 2.49 | 0.8 | 7.48 |
|  |  | Markazi | Female | 3.82 | 1.43 | 9.85 |
|  |  |  | Male | 1.59 | 0.4 | 6.16 |
|  |  | Mazandaran | Female | 6.58 | 2.15 | 18.41 |
|  |  |  | Male | 3.2 | 0.81 | 11.88 |
|  |  | Qazvin | Female | 1.88 | 0.47 | 7.25 |
|  |  |  | Male | 2.35 | 0.76 | 7.08 |
|  |  | Qom | Female | 1.04 | 0.15 | 7.06 |
|  |  |  | Male | 0.76 | 0.11 | 5.24 |
|  |  | Semnan | Female | 1.45 | 0.46 | 4.48 |
|  |  |  | Male | 2.34 | 1.04 | 5.2 |
|  |  | Sistan and Baluchistan | Female | 0.96 | 0.13 | 6.55 |
|  |  |  | Male | 6.03 | 2.85 | 12.29 |
|  |  | Tehran | Female | 1.89 | 0.27 | 12.19 |
|  |  |  | Male | 15.65 | 8.74 | 26.43 |
|  |  | Yazd | Female | 1.89 | 0.7 | 5.03 |
|  |  |  | Male | 0.79 | 0.2 | 3.14 |
|  |  | Zanjan | Female | 0.61 | 0.09 | 4.27 |
|  |  |  | Male | 0.8 | 0.2 | 3.16 |
| Poison | | Azerbaijan, East | Female | 24.36 | 3.37 | 74.82 |
|  |  |  | Male | 28.56 | 3.71 | 80.56 |
|  |  | Isfahan | Female | 23.83 | 3.28 | 74.28 |
|  |  | Khuzestan | Male | 35.83 | 5.16 | 85.14 |
|  |  | Kohgiluyeh and Boyer-Ahmad | Male | 6.99 | 0.77 | 42.07 |
|  |  | Mazandaran | Female | 27.57 | 3.98 | 77.75 |
|  |  | Qazvin | Female | 12.16 | 1.5 | 55.77 |
|  |  | Qom | Female | 12.07 | 1.49 | 55.54 |
|  |  | Tehran | Male | 28.62 | 3.72 | 80.61 |
| Scorpion or snake bite | | Khorasan, North | Male | 14.48 | 1.44 | 66.21 |
|  |  | Khorasan, Razavi | Male | 57.35 | 10.46 | 93.93 |
|  |  | Kurdistan | Male | 28.17 | 3 | 83.27 |
| Shock | | Isfahan | Male | 79.94 | 26.51 | 97.78 |
|  |  | Sistan and Baluchistan | Male | 20.06 | 2.22 | 73.49 |
| Hit | | Alborz | Female | 1.75 | 0.25 | 11.4 |
|  |  |  | Male | 0.84 | 0.12 | 5.74 |
|  |  | Ardabil | Female | 1.02 | 0.14 | 6.89 |
|  |  |  | Male | 0.91 | 0.23 | 3.59 |
|  |  | Azerbaijan, East | Female | 7.21 | 2.75 | 17.6 |
|  |  |  | Male | 3.51 | 1.33 | 8.95 |
|  |  | Azerbaijan, West | Female | 1.8 | 0.25 | 11.66 |
|  |  |  | Male | 5.07 | 2.31 | 10.77 |
|  |  | Bushehr | Female | 3.08 | 1.44 | 6.49 |
|  |  |  | Male | 1.7 | 0.84 | 3.41 |
|  |  | Chahar Mahaal and Bakhtiari | Female | 2.31 | 0.94 | 5.54 |
|  |  |  | Male | 1.93 | 0.99 | 3.72 |
|  |  | Fars | Female | 3.61 | 0.91 | 13.26 |
|  |  |  | Male | 6.2 | 3 | 12.38 |
|  |  | Gilan | Female | 1.83 | 0.26 | 11.84 |
|  |  |  | Male | 3.5 | 1.33 | 8.91 |
|  |  | Golestan | Female | 3.65 | 1.36 | 9.44 |
|  |  |  | Male | 1.66 | 0.62 | 4.36 |
|  |  | Hamadan | Female | 1.95 | 0.48 | 7.55 |
|  |  |  | Male | 3.7 | 1.84 | 7.31 |
|  |  | Hormozgan | Female | 2.63 | 0.65 | 9.95 |
|  |  |  | Male | 1.73 | 0.56 | 5.24 |
|  |  | Ilam | Female | 0.91 | 0.22 | 3.6 |
|  |  |  | Male | 1.06 | 0.44 | 2.54 |
|  |  | Isfahan | Female | 8.95 | 3.8 | 19.67 |
|  |  |  | Male | 10.88 | 6.46 | 17.73 |
|  |  | Kerman | Female | 9.73 | 4.14 | 21.2 |
|  |  |  | Male | 3.69 | 1.39 | 9.39 |
|  |  | Kermanshah | Male | 1.31 | 0.42 | 3.99 |
|  |  | Khorasan, North | Male | 0.87 | 0.32 | 2.3 |
|  |  | Khorasan, Razavi | Female | 7.56 | 2.89 | 18.38 |
|  |  |  | Male | 14.9 | 9.56 | 22.48 |
|  |  | Khorasan, South | Male | 1.08 | 0.44 | 2.6 |
|  |  | Khuzestan | Female | 7.1 | 2.71 | 17.37 |
|  |  |  | Male | 3.52 | 1.33 | 8.98 |
|  |  | Kohgiluyeh and Boyer-Ahmad | Female | 1.79 | 0.66 | 4.75 |
|  |  |  | Male | 1.29 | 0.57 | 2.87 |
|  |  | Kurdistan | Female | 0.85 | 0.12 | 5.84 |
|  |  |  | Male | 2.6 | 1.17 | 5.71 |
|  |  | Lorestan | Female | 3.67 | 1.37 | 9.48 |
|  |  |  | Male | 2 | 0.74 | 5.28 |
|  |  | Markazi | Male | 1.3 | 0.42 | 3.98 |
|  |  | Mazandaran | Female | 3.59 | 0.9 | 13.21 |
|  |  |  | Male | 5.23 | 2.37 | 11.11 |
|  |  | Qazvin | Male | 1.28 | 0.41 | 3.9 |
|  |  | Qom | Female | 1.78 | 0.44 | 6.9 |
|  |  |  | Male | 1.67 | 0.63 | 4.39 |
|  |  | Semnan | Female | 1.33 | 0.42 | 4.1 |
|  |  |  | Male | 1.9 | 0.98 | 3.65 |
|  |  | Sistan and Baluchistan | Female | 3.61 | 1.34 | 9.32 |
|  |  |  | Male | 1.71 | 0.64 | 4.49 |
|  |  | Tehran | Female | 18.31 | 10.26 | 30.53 |
|  |  |  | Male | 11.48 | 6.83 | 18.66 |
|  |  | Yazd | Male | 0.85 | 0.32 | 2.26 |
|  |  | Zanjan | Male | 0.65 | 0.21 | 2.01 |
| Suicide | | Isfahan | Female | 65.73 | 10.69 | 96.85 |
|  |  | Lorestan | Female | 34.27 | 3.15 | 89.31 |
|  |  |  | Male | 35.98 | 3.39 | 90 |
|  |  | Tehran | Male | 64.02 | 10 | 96.61 |
| Traffic | | Alborz | Female | 2.87 | 0.72 | 10.74 |
|  |  |  | Male | 3.15 | 1.32 | 7.31 |
|  |  | Ardabil | Female | 2.17 | 0.7 | 6.57 |
|  |  |  | Male | 0.94 | 0.3 | 2.89 |
|  |  | Azerbaijan, East | Female | 2.96 | 0.74 | 11.03 |
|  |  |  | Male | 3.15 | 1.32 | 7.31 |
|  |  | Azerbaijan, West | Female | 2.93 | 0.74 | 10.94 |
|  |  |  | Male | 4.36 | 2.11 | 8.82 |
|  |  | Bushehr | Female | 1.5 | 0.55 | 4 |
|  |  |  | Male | 2.2 | 1.29 | 3.72 |
|  |  | Chahar Mahaal and Bakhtiari | Female | 0.36 | 0.05 | 2.53 |
|  |  |  | Male | 1.1 | 0.52 | 2.3 |
|  |  | Fars | Female | 4.41 | 1.44 | 12.76 |
|  |  |  | Male | 8.95 | 5.41 | 14.44 |
|  |  | Gilan | Female | 3.14 | 0.79 | 11.68 |
|  |  |  | Male | 2.02 | 0.65 | 6.07 |
|  |  | Golestan | Female | 2.18 | 0.7 | 6.58 |
|  |  |  | Male | 3.42 | 1.9 | 6.09 |
|  |  | Hamadan | Female | 1.62 | 0.4 | 6.29 |
|  |  |  | Male | 2.32 | 1.1 | 4.81 |
|  |  | Hormozgan | Female | 4.08 | 1.54 | 10.4 |
|  |  |  | Male | 1.3 | 0.42 | 3.95 |
|  |  | Ilam | Male | 1.51 | 0.77 | 2.93 |
|  |  | Isfahan | Female | 13.18 | 7.07 | 23.27 |
|  |  |  | Male | 8.62 | 5.2 | 13.94 |
|  |  | Kerman | Female | 4.29 | 1.4 | 12.42 |
|  |  |  | Male | 6.13 | 3.24 | 11.32 |
|  |  | Kermanshah | Female | 0.72 | 0.1 | 4.95 |
|  |  |  | Male | 1.27 | 0.48 | 3.35 |
|  |  | Khorasan, North | Female | 1.88 | 0.77 | 4.51 |
|  |  |  | Male | 0.97 | 0.43 | 2.16 |
|  |  | Khorasan, Razavi | Female | 3.17 | 0.8 | 11.8 |
|  |  |  | Male | 9.08 | 5.49 | 14.65 |
|  |  | Khorasan, South | Female | 0.74 | 0.18 | 2.95 |
|  |  |  | Male | 0.94 | 0.42 | 2.09 |
|  |  | Khuzestan | Female | 8.8 | 3.72 | 19.4 |
|  |  |  | Male | 10.26 | 6.44 | 15.98 |
|  |  | Kohgiluyeh and Boyer-Ahmad | Female | 1.1 | 0.35 | 3.39 |
|  |  |  | Male | 1.26 | 0.63 | 2.52 |
|  |  | Kurdistan | Female | 2.19 | 0.7 | 6.62 |
|  |  |  | Male | 1.25 | 0.47 | 3.28 |
|  |  | Lorestan | Female | 4.85 | 2.15 | 10.55 |
|  |  |  | Male | 1.27 | 0.48 | 3.35 |
|  |  | Markazi | Female | 2.24 | 0.72 | 6.77 |
|  |  |  | Male | 2.23 | 1.06 | 4.61 |
|  |  | Mazandaran | Female | 4.67 | 1.52 | 13.48 |
|  |  |  | Male | 1.24 | 0.31 | 4.83 |
|  |  | Qazvin | Female | 2.2 | 0.7 | 6.64 |
|  |  |  | Male | 1.25 | 0.47 | 3.29 |
|  |  | Qom | Female | 2.88 | 1.08 | 7.49 |
|  |  |  | Male | 2.16 | 1.03 | 4.46 |
|  |  | Semnan | Female | 1.16 | 0.37 | 3.58 |
|  |  |  | Male | 0.63 | 0.24 | 1.69 |
|  |  | Sistan and Baluchistan | Female | 1.82 | 0.44 | 7.19 |
|  |  |  | Male | 4.6 | 2.73 | 7.66 |
|  |  | Tehran | Female | 13.24 | 7.1 | 23.37 |
|  |  |  | Male | 8.31 | 4.91 | 13.71 |
|  |  | Yazd | Female | 0.79 | 0.19 | 3.13 |
|  |  |  | Male | 2.31 | 1.36 | 3.91 |
|  |  | Zanjan | Female | 1.86 | 0.76 | 4.46 |
|  |  |  | Male | 1.79 | 0.98 | 3.22 |
